# Investigation, management and control of a maedi outbreak in Norway in 2019-2020

**DOI:** 10.1186/s13028-024-00749-7

**Published:** 2024-07-04

**Authors:** Grim Rømo, Johan Åkerstedt, Anne Bang Nordstoga, Anniken Jerre Borge, Helene Wisløff, Britt Gjerset, Siv Klevar, Mette Valheim, Irene Skei Mjømen, Elisabeth Schei-Berg, Synnøve Vatn, Annette Hegermann Kampen

**Affiliations:** 1 Norwegian Veterinary Institute, Elizabeth Stephansens vei 1, Ås, 1433 Norway; 2 Norwegian Food Safety Authority, Stensberggata 25/27, Oslo, 0170 Norway; 3 Animalia AS, Lørenveien 38, Oslo, 0585 Norway

**Keywords:** Disease elimination, Outbreak investigation, Ovine progressive pneumonia, Sheep, Small ruminant lentivirus, Surveillance, Visna-maedi virus infection

## Abstract

**Background:**

Visna-maedi is a notifiable disease in Norway, and eliminating the disease is a national goal. The import of sheep into Norway is very limited, and strict regulations apply to the movement of small ruminants between flocks and within defined geographical regions. Several outbreaks have occurred in the last 50 years, and the most recent before 2019 occurred in Trøndelag county in Central Norway in 2002. A national surveillance programme for small ruminant lentivirus infection exists since 2003.

**Results:**

In 2019, the national surveillance programme detected seropositive animals for small ruminant lentivirus in a sheep flock in Trøndelag. Based on the result of polymerase chain reaction analysis and histopathological findings, the Norwegian Food Safety Authority concluded the diagnosis of maedi. Further investigations detected maedi in eight additional sheep flocks in the same county. The flocks were placed under restrictions, and the authorities also imposed restrictions on 82 contact flocks. Sequencing of partial *gag* genes indicated that the virus in the current outbreak was related to the small ruminant lentivirus detected in the same area between 2002 and 2005.

**Conclusions:**

The outbreak investigation shows the need for sensitive and specific diagnostic methods, and an improved and more targeted surveillance strategy. It also demonstrates the risk of disease spreading between flocks through animal movements, and highlights the importance of biosecurity and structured livestock trade. In addition to allowing livestock trade only from flocks documented free from maedi, it may be necessary to monitor sheep flocks over many years, when aiming to eliminate maedi from the Norwegian sheep population.

**Supplementary Information:**

The online version contains supplementary material available at 10.1186/s13028-024-00749-7.

## Background

Visna-maedi is a chronic, progressive disease of sheep, caused by visna-maedi virus (VMV). Visna-maedi virus and caprine arthritis-encephalitis virus (CAEV) are both defined as small ruminant lentivirus (SRLV) in the *Retroviridae* family. Infection with VMV with respiratory manifestations is called maedi, while disease dominated by neurological signs is called visna [1]. Maedi has a long incubation period, normally up to three years [2, 3]. The main pathological changes are heavy lungs due to chronic interstitial pneumonia and hypertrophy of smooth muscle [4]. The preferred diagnostic methods include enzyme-linked immunosorbent assay (ELISA) for antibody detection in serum and molecular methods like polymerase chain reaction (PCR) on blood or lung tissue [5]. The infection is persistent, no treatment or vaccine exists, and infected individuals can further spread VMV [6]. The infection manifests itself in clinical signs as restrained breathing, coughing and emaciation, and leads in general to reduced milk production and thereby reduced weight gain in lambs [7, 8].

The disease most commonly transmits between animals in close contact over time, especially during the indoor housing season, or from dam to offspring [1]. The virus usually spreads between flocks due to movement of live animals [9].

Visna-maedi was first described in 1939 in Iceland [2]. Today, VMV is widespread in most sheep-keeping countries except for Australia, Iceland, and New Zealand [7]. Apart from two cases in the 1970s [10], visna has not been registered in Norway. Hence, this article refers to disease caused by VMV as maedi.

Maedi is a notifiable disease in Norway. Suspicion or detection must be reported to the Norwegian Food Safety Authority (NFSA), which imposes restrictions on the movement of live animals. In the event of a positive diagnosis, individuals or the entire flock are ordered to be culled, depending on the prevalence of seropositive animals in the flock [11].

**Fig. 1:**
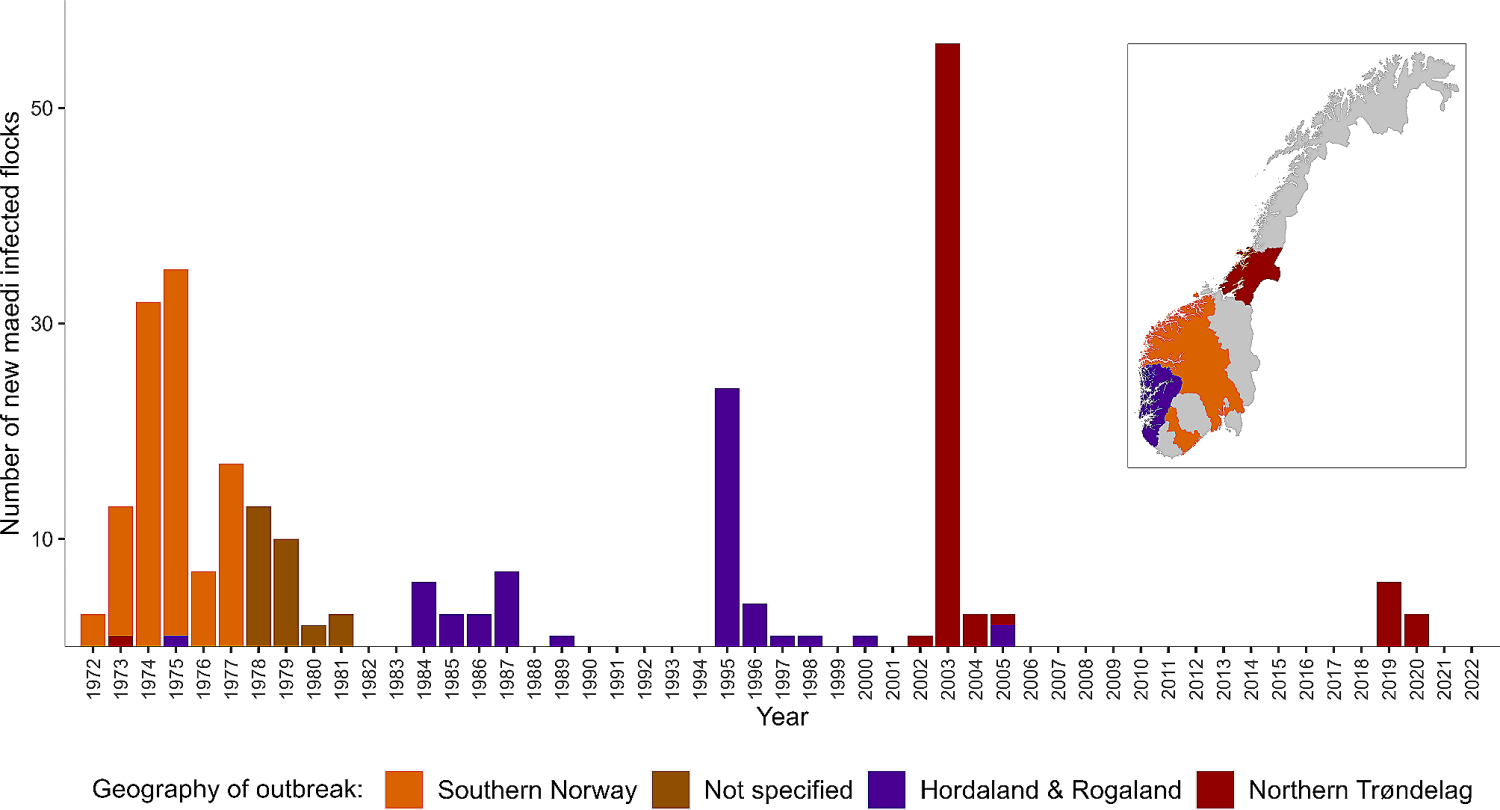
The number of detected maedi infected flocks from 1972 to 2022. The figure is modified after Krogsrud et al. [13], and thereafter updated with NVI journal system data and previous outbreak descriptions [10, 12]. The colours indicate the geographical location of affected flocks

Maedi was officially diagnosed for the first time in Norway in 1972 [10]. Outbreaks in several different parts of Norway followed during the 1970s and 80s (Fig. 1). In 1975, the veterinary authorities established a national control programme [12]. Massive sampling for serology led to the detection of 179 infected flocks and culling of thousands of animals. The number of annual detections of the disease gradually declined during the 1980s, and since no cases were detected from 1990 to 1994, the control programme ended in 1994.

In 1995, maedi reoccurred in Hordaland and Rogaland counties in Western Norway. Altogether, 29 positive flocks were discovered during 1995 and 1996 [Fig. 1 and 13]. The authorities initiated a regional surveillance programme for maedi in the two counties in 1997. Two more positive flocks were found, in 1998 and 2000, respectively [12].

In 2002, one seropositive sheep with typical pathology was detected at a farm in Trøndelag in Central Norway. The farm had 161 animals and played a major role as a supplier of breeding animals. The authorities imposed restrictions on around 250 contact flocks [12, 14]. Testing showed that among these, 61 flocks had seropositive sheep (Fig. 1). In flocks with more than two seropositive animals, all sheep were culled, while in flocks indicating infection of one or two individuals, only the seropositive animals and their offspring were culled [15]. Repeated serological testing on a yearly basis was obligated in both diagnosed flocks and contact flocks. The authorities lifted the restrictions in all flocks by 2007.

A national surveillance programme for small ruminant lentivirus infection exists since 2003. The surveillance programme for maedi has changed over the years regarding which flocks are included and the number of samples from each flock. During the years 2003 to 2008, breeding flocks constituted the target population for the surveillance programme. In the period 2010 to 2013, authorities obtained blood samples from sheep in randomly selected flocks. From 2014, the inspectors at the slaughterhouses conduct the sampling, ensuring a larger proportion of all flocks being tested each year [16].

In 2019, 14 years after the last outbreak, seropositive animals were detected in a sheep flock in Trøndelag as part of the ongoing surveillance programme [17]. The objective of this study was to describe this detection of maedi, and the investigation, management and control implemented in Norway during 2019 to 2022.

## Methods

### Study population

The study population included all sheep flocks in former Nord-Trøndelag county and some municipalities from the former Sør-Trøndelag, together forming the Northern part of the new county Trøndelag, hereafter called Northern Trøndelag. The area was chosen on the basis that maedi had been detected in this area previously and there had been very limited movement of animals out of this region. Existing flocks were serologically tested for antibodies against lentivirus during the outbreak investigation and monitoring between 2019 and 2022 (*n* = 675). The average flock size in this particular area was 80.7 breeding ewes in 2019, and 7.8% of the flocks had 200 sheep or more [18]. Some farmers are organised in local groups, in which a common pool of breeding rams circulate between several flocks in a so-called ram circle [19]. The ram circles consist of three to 20 flocks, and the rams mate with some ewes in each flock. In this geographical area, sheep are normally housed for aproximately six months during the winter season from before mating in November until some weeks after lambing. During summer, ewes and lambs are on pasture. The sheep are mainly of crossbred Norwegian white and produce meat and wool.

### Collection of samples and data

During the outbreak investigation, the NFSA checked farm records, traced contacts, and performed administrative follow-up of infected flocks and contact flocks. In addition, the NFSA collected blood samples for serological testing from all animals over one year of age in these flocks, at the farm or at slaughter. In flocks with seropositive animals, lungs with tracheobronchial and mediastinal lymph nodes and EDTA blood samples from selected cases were sent to the Norwegian Veterinary Institute (NVI) for pathological examination and PCR analysis for VMV.

### Diagnostics

The NVI analysed the serum samples for antibodies against SRLV using commercial ELISA kits. Initially, all samples were tested with ID Screen^®^ MVV/CAEV Indirect ELISA and/or ID Screen^®^ MVV/CAEV Indirect ELISA Verification kit (IDvet, Grabels, France). The diagnostic sensitivity and specificity of ID Screen^®^ MVV/CAEV Indirect ELISA in Norwegian sheep were estimated to be 99.3% and 99.1%, respectively [20]. ID Screen and ID verification tests are hereafter referred to as “IDvet” or “screening test” without further specification. IDvet positive samples were analysed in duplicates with the same test, and if still positive, they were further tested with IDEXX MVV/CAEV p28 Ab Verification Test (IDEXX Laboratories, Maine, USA), referred to as ”IDEXX” or “verification test”. The diagnostic sensitivity and specificity for this test in the same study were estimated to be 79.5% and 99.7%, respectively [20]. The tests and interpretation of the results as either positive, negative, or inconclusive, were performed according to the manufacturers’ instructions.

The weight of the lungs was registered, followed by macroscopic examination of the lung tissue and the tracheobronchial and mediastinal lymph nodes. Samples for histological examination and samples stored for subsequent PCR analysis were obtained from the cranial and caudal lobes, as well as the lymph nodes. The tissue samples were fixed in 10% neutral buffered formalin, processed by routine methods and stained with haematoxylin and eosin.

Peripheral blood mononuclear cells (PBMC) were isolated from the EDTA blood samples using Histopaque density gradient centrifugation (Sigma-Aldrich, Darmstadt, Germany). Genomic DNA was extracted from homogenised tissue samples of lung and lymph nodes or mononuclear cells using the NucliSense EasyMag^®^ protocol (Biomerieux, Craponne, France) according to the manufacturer’s recommendation. The samples were tested using a nested PCR targeting a 500 bp *gag* gene fragment of SRLV [21]. The PCR products obtained from the second PCR were analysed by Fragment Analyzer (Agilent, United Kingdom). Samples were purified using the ExoSAP-IT™ protocol (Thermo Fisher, Waltham, MA, USA) and sequenced on a 3500xl GA using a Big Dye™ Terminator v3.1 Cycle Sequencing Kit (Thermo Fisher, Waltham, MA, USA).

The obtained partial gag sequences were analysed with the MEGA-X package and compared with SRLV sequences from GenBank database with BLASTN, National Center for Biotechnology Information (NCBI).

### Epidemiological analysis

The NFSA recorded data from contact farms in their tracking and investigation module, which contained information of farm status of infection and imposed restrictions on the farms, contact farms, i.e., mainly movement of animals, animals on the farms, as well as sampling and results thereof. Information given in the submission forms and test results were registered in NVI’s journal system. Additional data sources included the Animal Health Register at NFSA (animal husbandry identity number, membership to ram circles) and the Register of Production Subsidies at the Norwegian Agriculture Agency (production sites, i.e., farms with geographical coordinates, and numbers of animals). The Norwegian Association of Sheep and Goat Farmers provided information of breeding rams. Data from the above data sources were imported, cleaned, interpreted, aggregated, and combined for production of descriptive statistics and analyses. All data management, analyses and visualisations were performed in R software for statistical computing and graphic [22].

### Definition of index farm and contact flocks

The first flock diagnosed with maedi in 2019, is referred to as the index farm, as the source of infection was unknown. According to the Norwegian guidelines for elimination of lentivirus infections, all flocks that had received sheep from, or moved sheep to, a flock with a maedi diagnosis during the last five years, were defined as contact flocks [11]. This included exchange of rams for breeding and shared inland pasture, but not rangeland or mountain pasture. When a ram from a ram circle had been used in, or sold to, a maedi positive flock, all flocks where the ram had been used earlier were defined as contact flocks. The NFSA also defined the index farm’s neighbouring flocks as contact flocks due to close contact between animals on pasture, shared transportation and jointly gathering of animals. In order to exclude CAEV infection in the index farm, all individuals in a neighbouring goat herd were tested.

### Diagnostic criteria for maedi

Heavy lungs (weight > 800 g) that did not collapse, enlarged tracheobronchial and mediastinal lymph nodes, and histopathological evidence of chronic interstitial pneumonia, were findings assessed to be consistent with maedi [4]. Flocks with PCR positive animals with sequencing results consistent with VMV were diagnosed with maedi.

Individuals with a positive result in the two different ELISA tests were defined as seropositive for SRLV. Contact flocks with one or more animals positive in both serological tests were diagnosed with maedi. In flocks diagnosed with maedi, all animals with a positive IDvet ELISA result were considered positive for maedi regardless of the test result in IDEXX.

In contact flocks not diagnosed with maedi, all animals with a positive IDvet ELISA result and negative test result in IDEXX were retested after three to four weeks. If still positive in IDvet and negative in IDEXX, the animal was slaughtered, and the lungs were obtained for pathological examination and PCR analysis.

### Restrictions and sanitation

Restrictions on any exchange of sheep and goats were imposed on flocks with maedi diagnosis and contact flocks. In flocks with more than 5% seropositive animals, all animals were slaughtered. In flocks with less than 5% seropositive animals, positive animals and their offspring or adopted lambs were slaughtered. These flocks got restrictions on movement for three years with yearly samplings [11].

Contact flocks that had received animals from a positive flock got restrictions for two years, with yearly sampling. Flocks that had delivered animals to a positive flock only got restrictions for one year with a minimum of two samplings with a one-year interval.

### Zone with regulations and a monitoring programme

To prevent spread of maedi out of Northern Trøndelag by livestock trade, the NFSA prioritised legislation, and a local regulation entered into force in September 2019 [23]. The authorities established a zone that included an area with 642 registered sheep flocks with 51.803 animals older than one year. The regulation enforced strict rules for trade in the zone, and prohibited sale of sheep and goats out of the zone.

The Ministry of Agriculture and Food decided to carry out monitoring of all sheep flocks in the zone, with the aim of detecting maedi if present in flocks not identified as contact flocks. A selection of 30 to 40 of the oldest animals were sampled depending on the flock size. In addition, rams, newly purchased animals and all animals for sale from the same flocks were sampled. The sampling was organised by the NFSA and carried out by private practitioners. The monitoring programme was repeated in the same area in 2021 to 2022 with up to 100 animals sampled per flock.

## Results

**Fig. 2:**
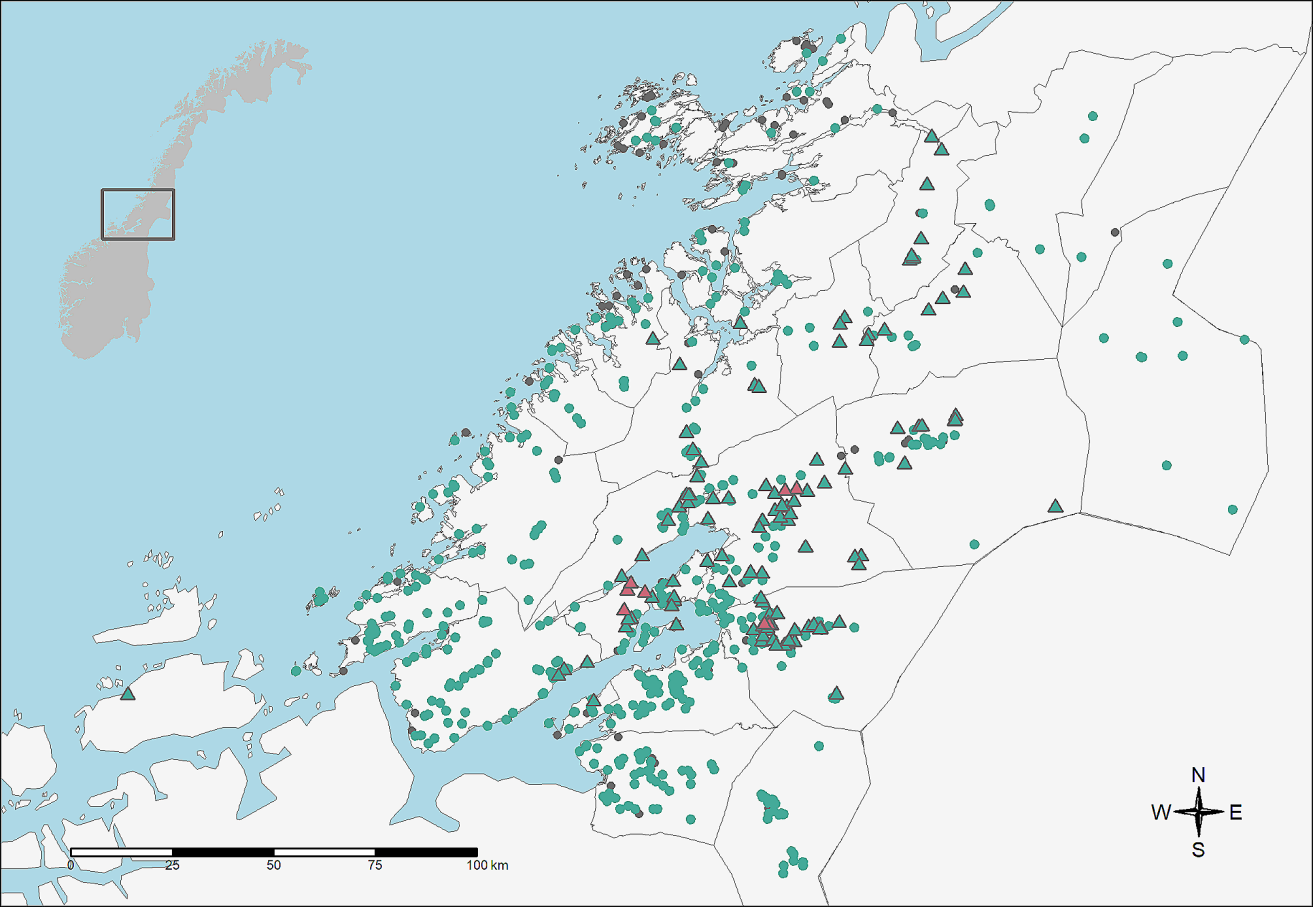
Sheep flocks involved in the maedi outbreak in Trøndelag in 2019 to 2020. Red triangles mark infected flocks. Green triangles mark negative contact flocks. Green dots mark monitored flocks within the zone of regulations. Grey dots represent other, not tested sheep flocks

Nine flocks got a maedi diagnosis in the described outbreak. The NFSA registered 136 contact flocks in addition to the index flock (Fig. 2) and placed 91 flocks under restrictions.

### Index flock

In March 2019, the NVI received four blood samples from a sheep flock with approximately 250 animals in Trøndelag county as a part of the national surveillance programme for SRLV. Three of the samples, taken from animals of four years or older, were positive in the screening test, while one sample was negative. Of the three positive samples, the verification test confirmed one sample as positive, but one was inconclusive and one negative.

The NFSA declared suspicion of maedi and collected follow-up blood samples from twenty-six animals, two years of age or older, in the same flock. Twelve additional animals were positive in the screening test. Four of these were positive also in the confirmation test, and were concluded seropositive for antibodies against SRLV.

Except for some coughing during the winter, the farmer had not observed symptoms of maedi in the flock. The NFSA considered all animals to be in good condition at sampling, except from one six-year-old emaciated ewe with an abnormal abdominal breathing (Additional file 1). This ewe and a seropositive ram with no clinical signs of maedi, were culled. The lungs of both animals showed macroscopic and histological changes consistent with maedi (Additional file 2). Small ruminant lentivirus was detected by PCR analyses of blood, lung and lymph node tissue from the ewe. Analysis of the proviral partial gag sequences confirmed that the virus belonged to the phylogenetic VMV genotype A group and showed genetic similarity with the virus detected in the same area during the years 2003–2005 [21]. The analysed sequence data was too limited for any further support on specific subtypes. Based on detection of antibodies against SRLV, detection of VMV and characteristic pathological changes in lungs and lymph nodes, the NFSA stated the maedi diagnosis in July 2019. A more detailed description of the pathological findings in the index herd is given in Additional file 2.

### Outbreak investigation

During the first year of outbreak investigation, a total of 10 370 blood samples from animals older than one year were collected from 116 flocks (Table 1). The NFSA registered 136 contact flocks. Twenty-one contact flocks were not sampled, of which eighteen flocks had terminated their sheep husbandry and the three remaining flocks were sampled later in 2020. The index flock and the other flocks in the area had mainly been recruiting rams from three different ram circles, which led to restrictions for flocks (*n* = 37) in all three ram circles. In addition to the index flock, the NFSA diagnosed maedi in four contact flocks (3.4%) from the primary outbreak investigation. Two of the contact flocks had sheep with macroscopic and histopathological lung lesions consistent with maedi, and VMV was detected by PCR from blood or lung tissue (Table 2). All samples from the neighbouring goat herd were negative for antibodies against SRLV.

**Table 1:** Sheep flocks tested for maedi in the outbreak zone from 2019 to 2022

	Outbreak investigation June 2019 – June 2020	Retesting contact flocks July 2020 – June 2021	Retesting contact flocks July 2021 – June 2022	Retesting contact flocks July 2022 – May 2023	Monitoring Oct. 2019 – June 2021	Monitoring July 2021 – Nov. 2022	Total (unique flocks / animals)
No. of flocks*	642	617	585	577	642	585	717
Sampled flocks	116	81	37	63	483	423	675
Sampled animals (> 1 year old)	10 370	9 408	2 218	4 237	11 728	18 879	44 379

*Based on data from the Register of production subsidies for the current years

### Monitoring programme

In the monitoring programme in 2019 to 2020 an additional 11 728 blood samples were analysed, from 483 flocks that were not defined as a part of the outbreak (Table 1). Four flocks were discovered as infected with maedi as a result of the monitoring programme (0.8%). Three of these flocks had animals with macroscopic and histopathological changes consistent with maedi, and VMV was detected by PCR (Table 2). In one of these flocks with a high prevalence of seropositive animals, goats were also present. One of the goats was seropositive, but neither VMV nor CAEV were found by PCR analysis. The positive flocks detected in the monitoring programme and their contacts were included in the investigation of the outbreak.

In addition the monitoring programme revealed CAEV infection in a flock with both sheep and goats. The virus was confirmed with PCR and sequencing to be of CAEV-like genotype C [21], thus the flock was not considered part of the maedi outbreak and is not listed in the tables and figures in this article.

In the monitoring programme in 2021 to 2022, 18 879 blood samples from 423 flocks were collected. All samples were concluded negative (Table 1).

### Contingency response

The six flocks with a large proportion of infected animals were slaughtered within a few months after the diagnosis, while in the three low-prevalence flocks, only the seropositive animals and their offspring and adopted lambs were slaughtered. The NFSA tested 82 contact flocks with negative results and did not diagnose maedi in any new flock after January 2020. By summer 2023, restrictions had been lifted in all contact flocks.

## Discussion

Nine flocks were diagnosed with maedi in the 2019–2020 outbreak. Thus, this outbreak was small compared to previous outbreaks in Norway [12, 13]. The total number of flocks involved in the 2019–2020 outbreak in Trøndelag (*n* = 137) reflects the fact that most Norwegian sheep flocks have contact with other flocks, mainly through movements of live animals. Only one contact flock outside Northern Trøndelag was registered. The legislation for movement of sheep in Norway is strictly precautious. Movement of ewes between flocks is generally not allowed. Transfer of rams across county borders requires a veterinary certificate with specific testing for SRLV, and movement between four defined small ruminant regions of Norway is prohibited [24]. Legislation regulating movement of sheep has been crucial for limiting spread of several infectious diseases in sheep and goats in Norway [25–28]. Hence, strict regulations are imposed and extensive measures taken when VMV is detected.

Sequencing and phylogenetic analysis of partial *gag* genes showed that the virus in the described outbreak in Trøndelag probably had circulated undetected in the population in the same area for 14 years. The testing regime used in 2002 to 2005 had lower sensitivity than the current regime. It is therefore possible that a sheep in an infected low-prevalence flock with slaughter of single seropositive animals, or a sheep in a contact flock, could have been false seronegative at that time [29]. In 2002 to 2005 the NFSA followed up contact flocks and infected flocks by yearly sampling for two or three years, respectively. This is a shorter follow-up time than in the 1990s, when infected flocks were followed for four years with a total of five rounds of testing [13]. This raises concerns whether one to two years of follow-up in contact flocks, and three years in infected flocks with selective slaughter in the 2019–2020 outbreak, are sufficient. Future monitoring in the area and/or sampling in selected flocks, may be necessary.

Another possible cause of spread is that the virus had persisted in a flock that was not identified as a contact flock in the 2002–2005 outbreak, and was therefore not sampled. Tracking contact flocks normally included contacts five years back in time. However, not all farmers had complete records of animal movements. Therefore, during the 2019–2020 outbreak, it was decided to screen all flocks, sampling up to 40 animals in each flock, in the zone with regulations. Monitoring of flocks in Northern Trøndelag led to the detection of four positive flocks that had not been identified as contact flocks and consequently not sampled. These flocks would not have been detected by tracking of contacts according to the legislation. This illustrates the importance of establishing zones with restrictions on movement, and sampling all animals in such zones. A second and more sensitive monitoring programme, sampling up to 100 animals older than one year in each flock, started in 2021. By the end of 2022, still no samples had been concluded as seropositive in this programme.

Elimination of maedi is a national goal in Norway. To achieve this, sensitive diagnostic methods are crucial. Low viral load in infected animals limits the performance of molecular methods (PCR). Hence maedi diagnostic is mainly performed using serology [6, 30]. The screening test used in the outbreak investigation has a reported sensitivity of 99.3% (95% confidence interval 97.4–100%) and a specificity of 99.1% (98.0-99.8%) [20]. This means that false positive samples could occur in up to 2% of the samples. Complementary analysis with a verification test, with a specificity of close to 100%, but with a much lower sensitivity (79.5%) [20], would confirm seropositivity in some infected flocks, but not all. False positive results pose a major challenge when the consequences of a maedi diagnosis are large, including limited livestock trade and halted breeding. On the other hand, without restrictions and follow-up sampling, potential positive flocks may be overlooked, and VMV may persist. These challenges in the management of the outbreak show the importance of both sensitive and specific diagnostic methods [29].

In countries where maedi is endemic, studies have shown that lambs from seronegative ewes weigh 0.3–3 kg more by weaning than lambs from seropositive ewes [8] and that seronegative ewes weaned 0.11 more lambs per ewe-lambing than seropositive ewes [31]. Elimination of caprine arthritis encephalitis, another SRLV infection, from Norwegian goat herds was calculated to be economically favorable within an average of ten years [32]. In addition to the economic costs of the disease, respiratory problems, weight loss and susceptibility for other infections result in reduced animal welfare [8, 33].

Having no known cases of maedi during 2006–2018 in spite of an ongoing surveillance in all counties, shows that the prevalence of maedi in Norway has been very low for years [34]. Furthermore, the import of sheep to Norway is very limited [35]. Eliminating maedi in Norway may therefore be possible.

In Norway, there is a tradition for close cooperation between the animal health authorities and the livestock industry on disease prevention and elimination. Bovine virus diarrhoea, paratuberculosis, and ovine footrot are recent examples of animal disease elimination programmes [36–38]. This work requires constructive communication and cooperation between farmers, authorities and the NVI or other diagnostic laboratories. In the beginning of the outbreak, the NFSA established a working group, and the stakeholders and industry also got the opportunity to participate in the management of the outbreak. The NFSA revised the guidelines for elimination of SRLV infection after the detection of maedi in 2019. The main changes were more precise diagnostic criteria and shorter duration of restrictions for some categories of contact flocks. Representatives from the sheep industry participated in this work, securing anchoring and trust by the sheep farmers. Both the livestock industry organisations and the individual farmers were helpful with organising sampling of sheep in each flock. The involvement of the local veterinarians in sampling sheep flocks for the monitoring programme was important for implementation of the screening. In order to increase compliance, it is crucial to have economic compensation for farmers who are affected [39, 40]. Since maedi is a notifiable disease in Norway, producers who are required to slaughter animals receive compensation from the authorities, according to rates given in the legislation [41].

The current outbreak was detected by seropositive samples in the surveillance programme. Given that few sheep revealed clear clinical symptoms and no pathological changes were reported from slaughterhouses, the outbreak could probably have continued if not detected in the ongoing surveillance. Although seven of the nine positive flocks had been sampled in the surveillance programme for maedi in the period 2006 to 2018, none of them were detected at that time. This could be because they were not yet infected or the prevalence in the flock was low. Because only a limited number of individuals from each flock were sampled in the surveillance programme, low-prevalence flocks had a relatively low probability of being detected. Some of the high-prevalence flocks were quite small (Table 2), and the probability of animals from these flocks being sampled at the slaughterhouse was also relatively low.

**Table 2:** Results of the outbreak investigations in the nine flocks where maedi was diagnosed

Reason for sampling	Flock	Pathology	PCR result	Seropositive (tested animals)	Apparent prevalence (%)
Surveillance	A	+	+	67 (205)	32.7
Contact flock	B	-	-	4 (302)	1.3
Contact flock	C	+	+	15 (17)	88.2
Contact flock	D	*	*	2 (131)	1.5
Contact flock	E	+	+	50 (63)	79.4
Monitoring	F	+	+	35 (38)	92.1
Monitoring	G	+	+	41 (45)	91.1
Monitoring	H	-	-	1 (77)	1.3
Monitoring**	I	+	+	37 (56)	66.1

* No samples received

** Contact flock to one of the flocks detected in the monitoring programme

Clusters of positive flocks may possibly exist in other parts of Norway where cases of maedi have occurred in the past. A more risk-based selection of samples, focusing on flocks in defined geographical areas and sampling of older animals [29], together with an increase in the number of sampled sheep, might improve the sensitivity of the programme. Critical evaluation of the surveillance programme and steps to target and optimise future surveillance are called for.

Except for the emaciated ewe in the index flock (Additional file 1), the NFSA registered no clinical symptoms of maedi when taking blood samples in infected flocks. In retrospect, one of the farmers reported that many ewes had lost weight despite increased feeding, and some farmers reported sporadic coughing, which might as well be due to lung worm infestation or other respiratory infections. However, in some of the flocks, typical pathological lesions for maedi were found *post mortem* in a large proportion of the slaughtered animals. Reduced lung capacity, respiratory distress and coughing due to these lesions are likely to have occurred. Moreover, the pathological changes in some of the animals were clearly chronic, and lungs with pathological changes from other animals from these flocks have probably gone undetected at meat inspection at slaughter. *Post-mortem* examinations of lungs in slaughterhouses have been improved by courses on macroscopic maedi lesions. Older animals, or animals in poor condition are, however, not necessarily sent to slaughter.

Despite strict legislation, the outbreak illustrates that the industry is vulnerable. Many sheep flocks have contacts that would need to be investigated in case of a disease outbreak, and some livestock movements are insufficiently registered. Purchase of rams from several ram circles substantially increases the number of contact flocks. Increased awareness of the risks posed by movement of animals and a system for safer livestock trade would reduce risk of disease spread and minimise the consequences of an outbreak.

## Conclusions

The nature of maedi, with its slow progression, vague symptoms and delayed seroconversion, makes detection of infected animals difficult. Interpretation of ambiguous serological results poses challenges to the outbreak management and shows the need for sensitive and specific diagnostic methods. The surveillance strategy for maedi in Norway should be evaluated.

Analysis of partial *gag* gene sequences from the 2019–2020 outbreak in Northern Trøndelag indicated genetic relationship to a VMV strain detected in the same area during a previous outbreak in 2002 to 2005. An initiated monitoring programme, aiming to detect maedi if present in flocks not identified as contact flocks, led to detection of four infected flocks, in addition to the five flocks detected in the primary outbreak investigation. Monitoring all sheep flocks in the area repeatedly over many years, would be crucial for documentation of freedom, or early detection of reoccurrence of maedi.

The maedi outbreak investigation in 2019 to 2020 demonstrates the risk of disease spreading between flocks by animal movements, and highlights the importance of biosecurity and structured livestock trade. It might be necessary to allow livestock trade only from flocks documented free from maedi, when aiming to eliminate maedi from the Norwegian sheep population.

### Electronic supplementary material

Below is the link to the electronic supplementary material.

Additional file 1: Ewe from the index flock with clinical signs of maedi.

Additional file 2: Pathological findings in the index flock.

## Data Availability

The datasets used and analysed during the current study are available from the corresponding author on reasonable request.

## Declarations

### Prior publication

Data included in this article have previously been published in the Proceedings of the digital International Sheep Veterinary Conference November 24th 2021, the conference of the International Society for Veterinary Epidemiology and Economics in Halifax, Canada, August 12th 2022 and in the Norwegian veterinary journal no. 2, 2021. Pictures included in Additional file 2 have previously been published in the Norwegian veterinary journal no. 7, 2019.

## Abbreviations

BLASTN: Nucleotide basic local alignment search tool
CAEV: Caprine arthritis-encephalitis virus
EDTA: Ethylenediaminetetraacetic acid
ELISA: Enzyme-linked immunosorbent assay
NCBI: National Center for Biotechnology Information
NFSA: Norwegian Food Safety Authority
NVI: Norwegian Veterinary Institute
PBMC: Peripheral blood mononuclear cells
PCR: Polymerase chain reaction
SRLV: Small ruminant lentivirus
VMV: Visna-maedi virus
