# Supplementary material for: Investigation, management and control of a maedi outbreak in Norway in 2019-2020
Source: Acta Vet Scand. 2024 Jul 4;66:28. doi: 10.1186/s13028-024-00749-7 (PMC11225221; doi:10.1186/s13028-024-00749-7)
Supplement: Supplementary file 2 — Additional file 2: Pathological findings in the index flock. [file 13028_2024_749_MOESM2_ESM.docx]

**ADDITIONAL FILE 2**

**Pathological findings in the index flock**

***Additional file 2 to the paper:***

**Investigation, management and control of a maedi outbreak in Norway in 2019-2020**

**Grim Rømo^1*^, Johan Åkerstedt^1^, Anne Bang Nordstoga^1^, Anniken Jerre Borge^1^, Helene Wisløff^1^, Britt Gjerset^1^, Siv Klevar^1^, Mette Valheim^1^, Irene Skei Mjømen^2^, Elisabeth Schei-Berg^2^, Synnøve Vatn^3^, Annette Hegermann Kampen^1^**

***Pathological findings in the index flock***

The Norwegian Food Safety Authority euthanized a six-year old emaciated and dyspnoeic ewe (Figure 1), and a five-year old seropositive ram with no observed clinical signs of maedi. Lungs from both animals were sent to the NVI for pathological examination and virus detection and identification.

The lungs of the ewe and the ram weighed 1192 and 1064 grams respectively, and both lungs were moderately firm and did not collapse. In the ewe’s lung there were dark red irregular areas both in the cranial and caudal lobes (Figure 2). There were small areas with emphysema in the cranial lobes, and scattered subpleural nodules in the caudal lobes, probably lesions caused by lung worms. The tracheobronchial and mediastinal lymph nodes were enlarged.

Histological examination of the lung from the ewe showed severe hypertrophy of smooth muscle and peribronchiolar and perivascular infiltration of mononuclear cells (Figures 3 and 4). In some areas the lung tissue was completely condensed. Similar findings, but milder, were observed in the lung from the ram.

***Figures***


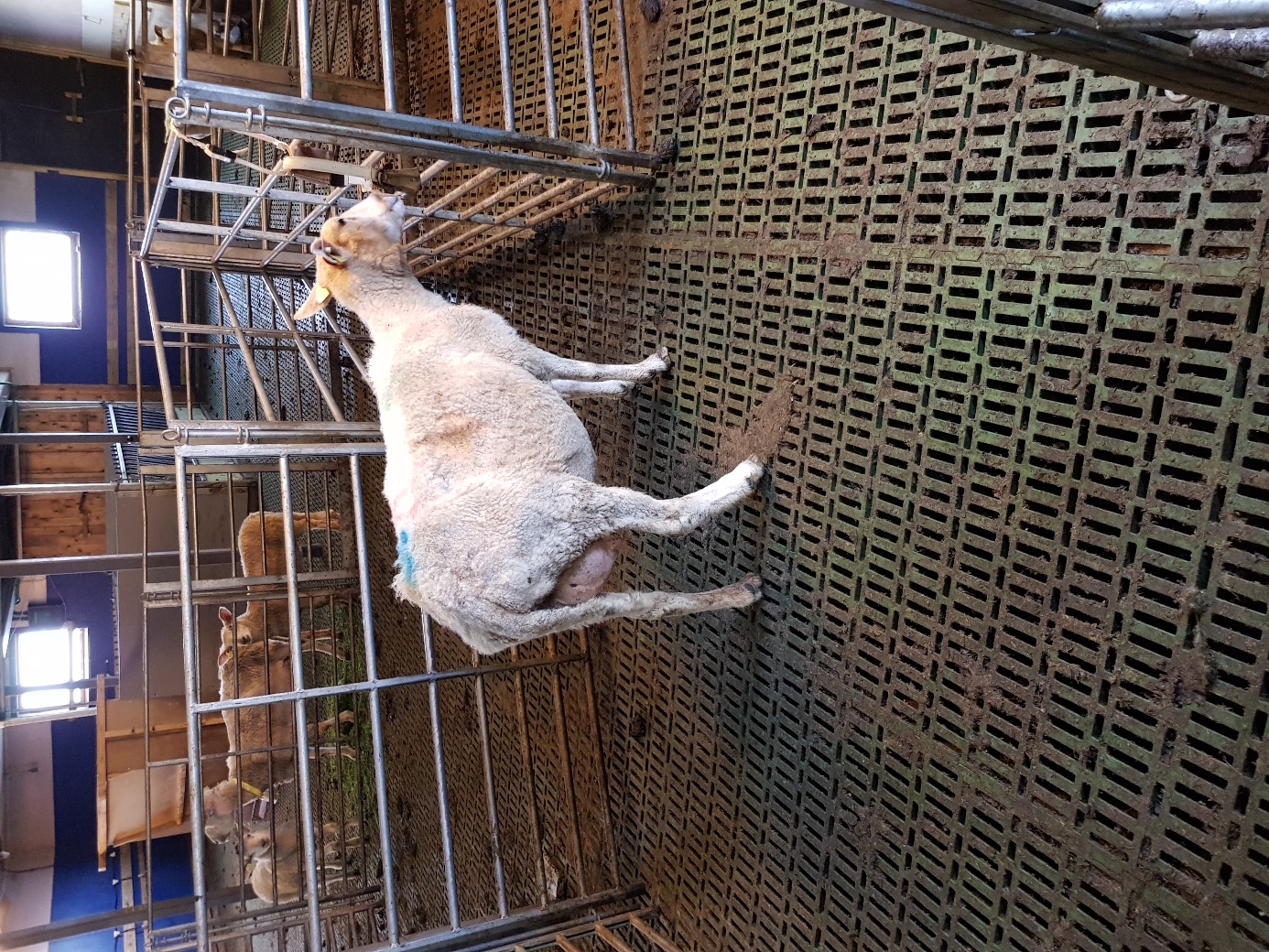


Figure 1. A six-year old emaciated and dyspnoeic ewe diagnosed with maedi.


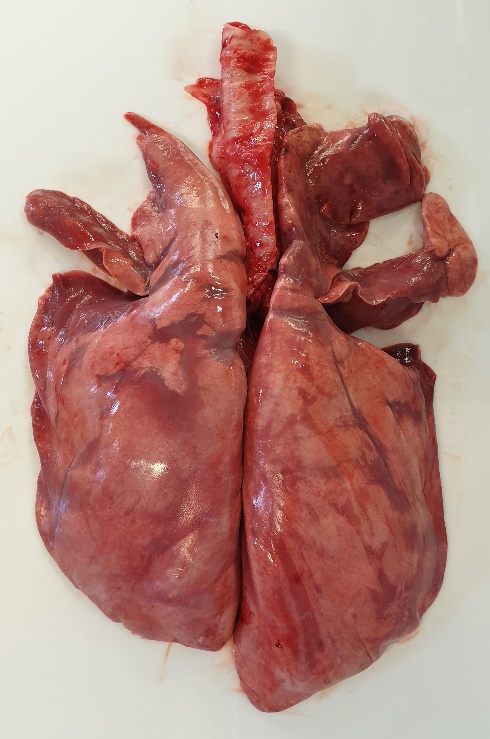

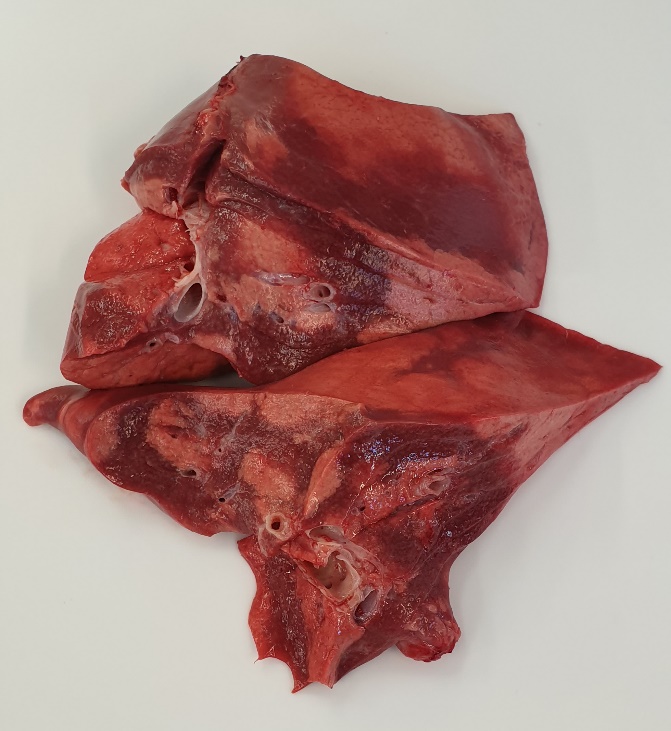


Figure 2. Lung from a ewe with maedi. The lesions consisted of pale multifocal to more diffuse, rubbery to firm tissue, alternating with irregular, dark red and atelectatic areas.


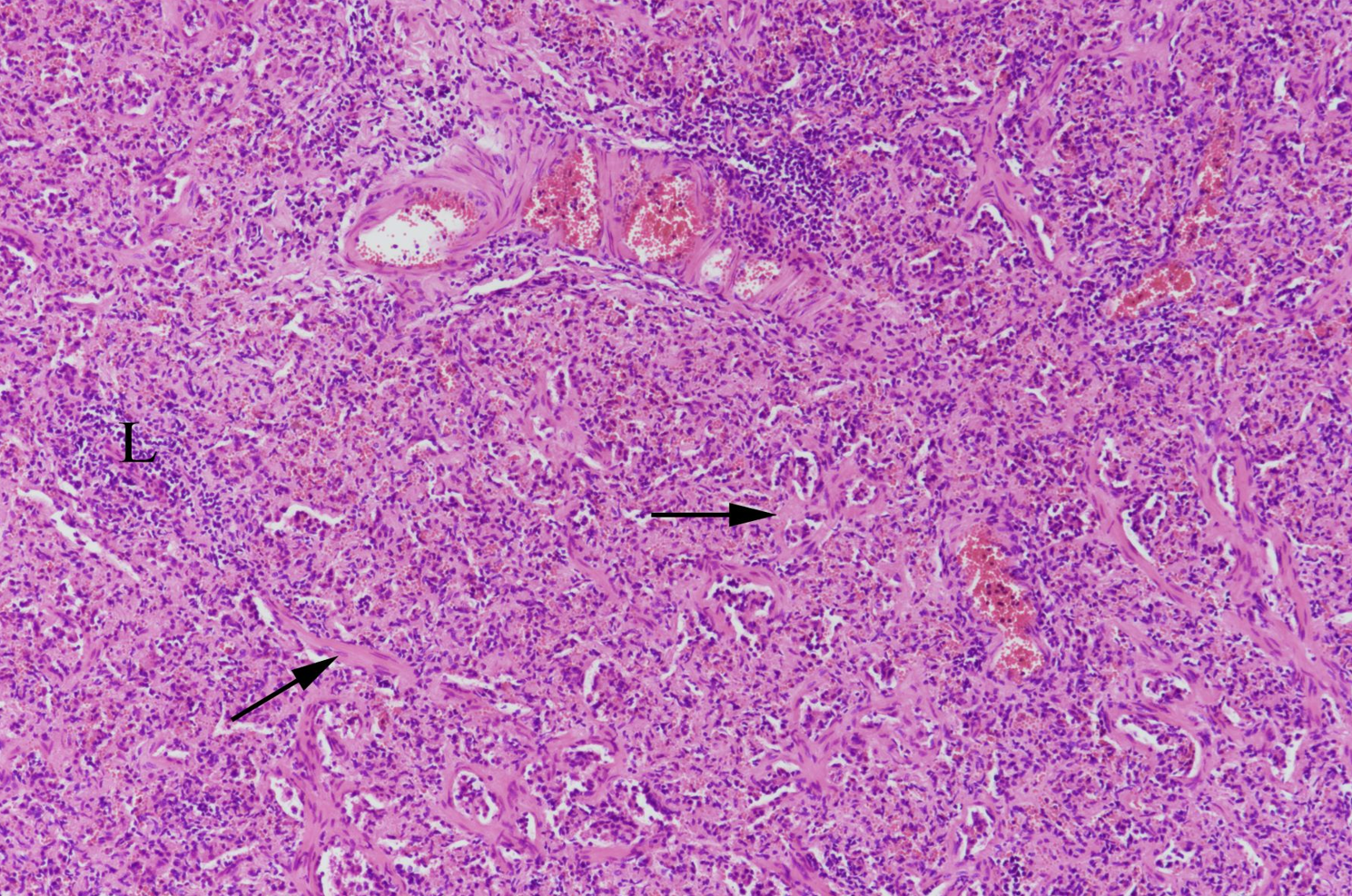


Figure 3. Histological section of lung from a ewe with maedi, HE-staining. Severe hypertrophy of smooth muscle in thickened alveolar septa (arrows), and peribronchiolar and perivascular infiltration of mononuclear cells (L).


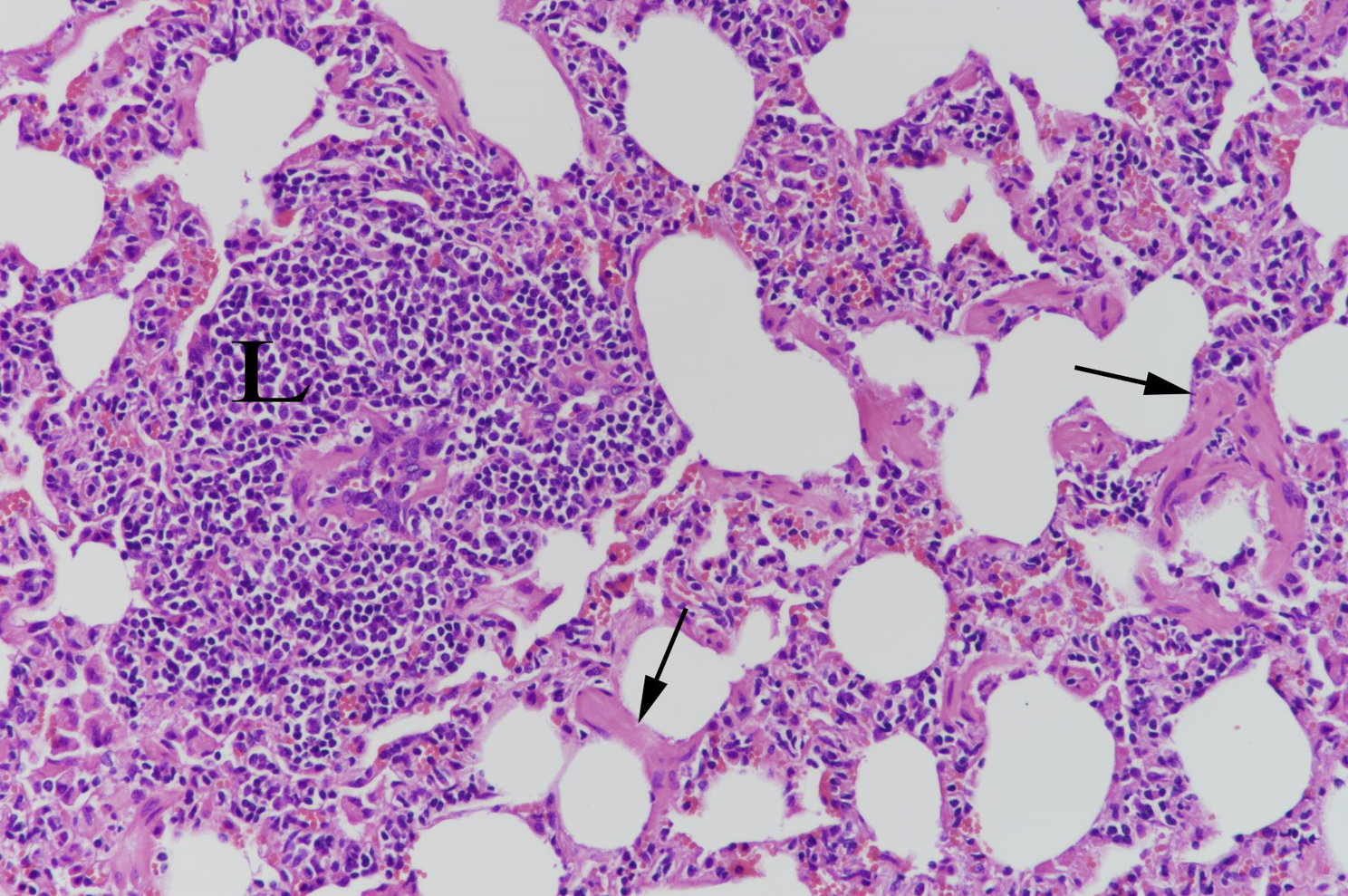


Figure 4. Histological section of lung from a ewe with maedi, HE-staining. Hypertrophy of smooth muscle (arrows), and peribronchiolar and perivascular infiltration of mononuclear cells (L).
